# Mentorship and self-efficacy are associated with lower burnout in physical therapists in the United States: a cross-sectional survey study

**DOI:** 10.3352/jeehp.2023.20.27

**Published:** 2023-09-27

**Authors:** Matthew Pugliese, Jean-Michel Brismée, Brad Allen, Sean Riley, Justin Tammany, Paul Mintken

**Affiliations:** 1 Department of Physical Therapy, Hospital for Special Surgery, New York, NY, USA; 2 Department of Rehabilitation Sciences, Center for Rehabilitation Research, Texas Tech University Health Sciences Center, Lubbock, TX, USA; 3 Hartford Healthcare Rehabilitation Network, Glastonbury, CT, USA; 4 Department of Physical Therapy, Hardin-Simmons University, Abilene, TX, USA; 5 Doctor of Physical Therapy Program, Graduate College of Health Sciences, Hawai‘i Pacific University, Honolulu, HI, USA; Hallym University, Korea

**Keywords:** Prevalence, Professional burnout, Psychological burnout, Surveys and questionnaires, United States

## Abstract

**Purpose:**

This study investigated the prevalence of burnout in physical therapists in the United States and the relationships between burnout and education, mentorship, and self-efficacy.

**Methods:**

This was a cross-sectional survey study. An electronic survey was distributed to practicing physical therapists across the United States over a 6-week period from December 2020 to January 2021. The survey was completed by 2,813 physical therapists from all states. The majority were female (68.72%), White or Caucasian (80.13%), and employed full-time (77.14%). Respondents completed questions on demographics, education, mentorship, self-efficacy, and burnout. The Burnout Clinical Subtypes Questionnaire (BCSQ-12) and self-reports were used to quantify burnout, and the General Self-Efficacy Scale (GSES) was used to measure self-efficacy. Descriptive and inferential analyses were performed.

**Results:**

Respondents from home health (median BCSQ-12=42.00) and skilled nursing facility settings (median BCSQ-12=43.00) displayed the highest burnout scores. Burnout was significantly lower among those who provided formal mentorship (median BCSQ-12=39.00, P=0.0001) compared to no mentorship (median BCSQ-12=41.00). Respondents who received formal mentorship (median BCSQ-12=38.00, P=0.0028) displayed significantly lower burnout than those who received no mentorship (median BCSQ-12=41.00). A moderate negative correlation (rho=-0.49) was observed between the GSES and burnout scores. A strong positive correlation was found between self-reported burnout status and burnout scores (r_rb_=0.61).

**Conclusion:**

Burnout is prevalent in the physical therapy profession, as almost half of respondents (49.34%) reported burnout. Providing or receiving mentorship and higher self-efficacy were associated with lower burnout. Organizations should consider measuring burnout levels, investing in mentorship programs, and implementing strategies to improve self-efficacy.

## Graphical abstract

[Fig f2-jeehp-20-27]

## Introduction

### Background/rationale

Burnout is an occupational and psychological syndrome characterized by mental and physical exhaustion caused by one’s professional life. The syndrome is associated with prolonged workplace stress, feelings of exhaustion, negativism, and reduced professional efficacy. It is pervasive in human service professions where a relationship is established between patients and clients with physical or emotional needs. The risk of developing burnout is nearly twice as high in physicians than in the general population. It is also widespread in the physical therapy profession, with one study reporting burnout in 53% of subjects [1,2].

The effects of burnout are staggering and can directly impact patients’ and providers’ well-being. Burned out physicians display poor communication, reduced teamwork, lower empathy, and non-adherence to treatment guidelines [3]. Additionally, burnout is associated with reduced patient satisfaction, suboptimal quality of care, increased medical errors, and adverse health consequences for providers [1,4-6]. Burnout has a significant economic impact as it is associated with increased practitioner turnover, absenteeism, reduced professional effort, job dissatisfaction, and increased odds of leaving a job [1,6]. However, research on the cost of burnout in the physical therapy profession in the United States is currently lacking [7].

Burnout has traditionally been defined by high emotional exhaustion, high depersonalization, and feelings of low accomplishment [8]. More recently, burnout has been characterized by subtypes associated with overload, lack of development, and neglect. This categorization is advantageous as it may help explain the underlying origins of the syndrome [8,9]. Overload is defined as disregarding personal health and well-being while pursuing work achievements. It is associated with high exhaustion levels, coping focused on active problem-solving, and increased work hours. Lack of development occurs when tasks are monotonous and under-stimulating, and is associated with coping involving distraction and cognitive avoidance. Neglect is related to low levels of perceived self-efficacy and is observed when individuals feel hopeless due to a lack of control and do not feel acknowledged for effort at work [10]. No study has investigated the associations of the modifiable factors of post-professional education, mentorship, professional organization membership, and self-efficacy with physical therapists’ burnout.

### Objectives

The purpose of this study was to determine: (1) the prevalence of burnout in physical therapists in the United States using a validated measurement tool called the Burnout Clinical Subtypes Questionnaire (BCSQ-12) and self-reports; and (2) the relationships between burnout dimensions and academic degree level, mentorship received, mentorship provided, continuing education coursework attendance, advanced professional certifications, professional organization membership, number of unique job roles, and self-efficacy level measured using the General Self-Efficacy Scale (GSES).

## Methods

### Ethics statement

This study protocol was exempted by the Texas Tech University Health Sciences Center Institutional Review Board (IRB-L#00000096).

### Study design

This was a cross-sectional survey study. It was described according to the STROBE statement (https://www.strobe-statement.org/).

### Setting

Data collection was conducted using the SurveyMonkey web-based survey (SurveyMonkey Inc.). Email lists were retrieved from a convenience sample from the state physical therapy boards of Florida, Michigan, Nebraska, North Carolina, Ohio, Oregon, Rhode Island, Texas, and West Virginia. The survey was administered over a 6-week period from December 2020 to January 2021.

### Participants

Participants were eligible if they were licensed physical therapists involved in active clinical practice and were excluded if they were unemployed, furloughed, retired, or not currently providing direct patient care.

### Variables

Respondents indicated the mentorship they provided (none, informal training, formal residency, or fellowship or student instructor) and received (none, informal training, formal residency, or formal fellowship training). The survey included questions regarding academic degree type (bachelor’s [PT], master’s [MPT], doctorate [DPT], or terminal academic degree [PhD, ScD, DSc, or EdD]), practice setting, care delivery model (in-person, virtual, or mixed), hours of continuing education, number of professional organization memberships, advanced physical therapy certifications, and unique job roles. Burnout and self-efficacy were also measured using publicly available instruments.

### Measurement

The GSES measured perceived self-efficacy during daily activities and stressful events [11]. It comprises 10 questions scored from 1 (not true at all) to 4 (exactly true) with a final composite score ranging from 10 to 40 and a higher score indicating greater self-efficacy. The GSES has been used in various research settings with internal consistency and Cronbach’s α ranging between 0.75 and 0.91 and test re-test reliability of r=0.67 [11]. Use of this tool was permitted by Dr. Ralph Schwarzer (Freie Universität Berlin).

The BCSQ-12 consists of 12 questions with 7 response options ranging from 1 (totally disagree) to 7 (totally agree) and items equally distributed between overload, lack of development, and neglect dimensions [9]. The minimum score is 12 and the maximum 84, with a higher score indicating greater burnout. The BCSQ-12 internal consistency has been reported to range from 0.53 to 0.7, and the kappa coefficient ranged from 65% to 82.5% [9]. Respondents were also asked, “do you currently consider yourself to be burned out as a physical therapist?” as part of the survey. Possible responses were limited to “yes” or “no.” This novel question has not been used in previous research, and its validity and reliability have not been established. Survey questionnaires are available from Supplement 1.

### Bias

No bias in participant selection was identified as a convenient sample of physical therapists was obtained.

### Study size

Sample size estimations were not made as the population characteristics and expected response rate were unknown.

### Statistical methods

Descriptive statistics were calculated using IBM SPSS ver. 27.0 (IBM Corp.) for the subjects’ characteristics, including age, sex, years of practice, and all variables. For ordinal and nominal data, non-parametric inferential statistics were used. All statistics were conducted using an alpha level <0.05. Kruskal-Wallis analysis of variance was used to determine differences between practice setting, care delivery model, academic degree type, mentorship received, and provided on burnout. Spearman’s rho was used to determine relationships between physical therapists’ continuing education level, advanced certification level, professional organization membership level, unique job roles, self-efficacy level (GSES), and burnout. Rank biserial correlation was used to determine the relationship between self-reported burnout and burnout (BCSQ-12).

## Results

### Participants

An invitation to participate in an internet survey was emailed to 80,112 physical therapists. A total of 22,603 individuals opened the email, 3,827 clicked the link to begin the survey, and 3,197 completed the survey. The responses from 384 surveys were excluded as participants reported being unemployed, furloughed, retired, or not currently involved in clinical practice. A sample of 2,813 practicing physical therapists was included in the data analysis (Datasets 1, 2). The sample included individuals from all 50 States, as respondents may have relocated or been licensed in multiple states. The respondents’ demographic characteristics are detailed in Tables 1–3.

### Main results

**Fig. 1.**
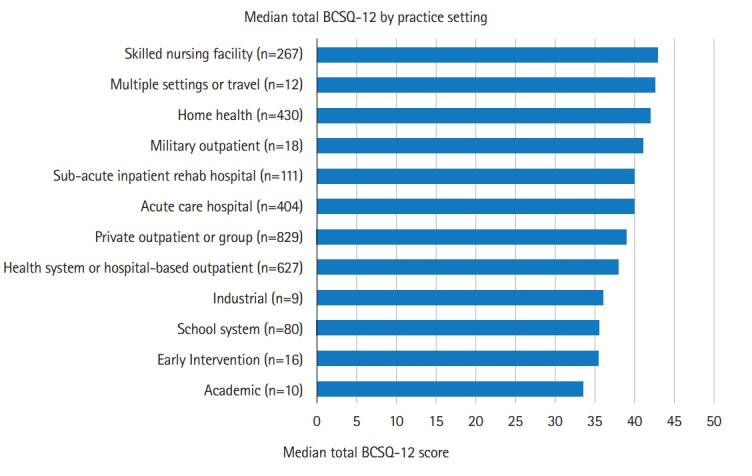
Median Burnout Clinical Subtypes Questionnaire (BCSQ-12) scores by physical therapy practice setting in the United States in 2021.

A significant main effect was observed for practice setting and burnout (BCSQ-12) (P<0.001; power=1.0000; η^2^=0.0320). Respondents in school systems (median=35.50) displayed significantly lower burnout than those in home health (P=0.0004) (median=42.00) and skilled nursing settings (P<0.0001) (median=43.00). Burnout was significantly lower in hospital-based outpatient clinics (median=38.00) when compared to home health (P<0.0001) (median=42.00) and skilled nursing settings (P<0.0001) (median=43.00). Similarly, private outpatient office or group practices had significantly lower burnout (median=39.00) than home health (P<0.0001) (median=42.00) and skilled nursing facility settings (P<0.0001) (median=43.00). Respondents practicing in acute care hospital settings (median=40.00) displayed significantly lower burnout than those in skilled nursing facility settings (P=0.0090) (median=43.00). Further information regarding burnout across practice settings is detailed in Table 2 and Fig. 1.

A significant main effect was observed for the academic degree and burnout (P<0.001; power=1.0000; η^2^=0.0182). Respondents with a terminal degree (P=0.0055) (median=33.50) and bachelor’s degree (P<0.0001) (median=38.00) displayed significantly lower burnout than those with a doctorate degree (median=40.00). The bachelor’s degree group (P=0.0049) (median=38.00) also displayed significantly lower burnout than the master’s degree group (median=39.00) (Table 3).

A significant main effect was displayed for the mentorship received and burnout (P<0.001; power=0.9624; η^2^=0.0059). Respondents who received formal mentorship (P=0.0028) (median=38.00) exhibited significantly lower burnout than those who received no mentorship (median=41.00). Similarly, a significant main effect was displayed for the mentorship provided and burnout (P<0.001; power=0.9842; η^2^=0.0070). Respondents who provided formal mentorship (P=0.0001) (median=39.00) displayed significantly lower burnout than those who provided no mentorship (median=41.00). Further information regarding mentorship experience and burnout is detailed in Table 4.

A moderate negative correlation was observed between GSES scores and neglect burnout scores (rho=-0.49). A strong positive correlation was displayed between self-reported burnout status (“Do you currently consider yourself to be burned out as a physical therapist?”) and burnout (BCSQ-12) (r_rb_=0.61). Weak or negligible positive or negative correlations were displayed between all other variables.

## Discussion

### Key results

Burnout is prevalent in the physical therapy profession. Providing or receiving mentorship and higher levels self-efficacy were associated with lower burnout.

### Interpretation

This may be one of the first studies to examine burnout and its association with modifiable factors in licensed physical therapists in the United States. A sample of 2,813 physical therapists was captured and was representative of physical therapists across the United States concerning age, gender, and race [12]. Approximately half of respondents (49.3%) reported that they were burned out. This finding is comparable to the rate of burnout reported by physical therapists (53%) and other healthcare practitioners, including physicians and nurses (35% to 54%) and medical residents (45% to 60%) [3,13].

A strong positive correlation was observed between self-reported burnout status and burnout (BCSQ-12). This finding is noteworthy as a burnout cutoff score, or minimal clinically important difference value, has not been established for the BCSQ-12. Asking healthcare providers a single question regarding burnout may be a quick and effective means to determine if they are burned out. Using additional burnout questionnaires, such as the BCSQ-12, may also be worthwhile to identify the specific dimensions of burnout that are impacted. Further research is warranted to determine if a single burnout question is a valid and reliable means to screen for burnout.

Receiving mentorship is a modifiable factor that may influence burnout experienced by physical therapists. Respondents who received formal mentorship displayed significantly lower burnout compared to those who received no mentorship. Additionally, those who received formal or informal mentorship displayed a lower self-reported burnout (45.1% and 47.3%, respectively) than those who received no mentorship (56.5%). Providing mentorship is another factor related to lower levels of burnout. Providing formal mentorship was associated with significantly lower burnout than not providing mentorship. Physical therapists who provided formal (47.1%) or informal (47.9%) mentorship exhibited lower self-reported burnout than those who provided no mentorship (56.7%).

Physical therapists may seek to receive or provide mentorship in formal or informal settings to develop skills and grow personally and professionally. Mentorship programs may improve self-confidence and increase professional competency in physical therapists. Investment into developing and promoting mentor and mentee relationships should be considered at both the individual and organizational levels. The financial and temporal costs of mentorship programs may be offset by improved job satisfaction, reduced turnover, absenteeism, greater professional effort, and lower burnout.

Self-efficacy is another factor that may influence burnout. A moderate negative correlation was observed between GSES scores and neglect burnout (rho=-0.49). Physical therapists may experience lower burnout when they feel capable of controlling challenging demands or situations [14]. It may be beneficial for employers to promote autonomy and flexibility and provide physical therapists with the necessary tools to handle job challenges. Physical therapists should consider measuring and reflecting upon their self-efficacy attitudes and beliefs and seek appropriate resources to augment their self-efficacy. Further research regarding the impact of self-efficacy training on physical therapist burnout is recommended.

The impact of academic degree type on burnout was less clear, as respondents who possessed terminal (PhD, ScD, DSc, EdD or equivalent) or bachelor’s degrees (PT) displayed significantly lower burnout than those with doctoral degrees (DPT). Changes in academic degree requirements may confound this finding, as a doctoral degree from an accredited institution is now required to enter the physical therapy profession. Obtaining a terminal academic degree may allow physical therapists to practice in an academic setting, increase involvement in mentorship, teaching, and research, and result in a more clearly defined career path. Further research on the effect of education on burnout should be conducted.

Disparities in burnout scores existed across physical therapy practice settings, with the skilled nursing facility and home health settings exhibiting the greatest burnout. Conversely, academic, early intervention, and school settings displayed the lowest burnout. Practice setting may be a factor that contributes to burnout as the rate does not appear to be uniform throughout the profession. Burnout is higher among nursing home physicians, and it has been reported that social interaction and work culture factors are important predictors of burnout [13,15]. Therefore, it may be plausible for the same individual to experience variable burnout based on their practice setting. The impact of changing practice settings is yet to be established and may be a strategy to mitigate burnout. Moreover, future investigation may reveal the specific factors of each practice setting, including home health and skilled nursing, that may be associated with the development of burnout.

Levels of continuing education, advanced certification, and professional organization membership were not associated with burnout. This finding is substantial, as advanced training or involvement may not protect physical therapists from experiencing burnout. Investment of time and financial resources into the attendance of continuing education seminars, specialty certificates, and professional organizational memberships may not be an effective means of preventing burnout. This finding contrasts with the impact of mentorship provided and received on burnout. The personal connection and relationship between a mentor and mentee may be a factor that influences burnout. Further investigation is required to better understand the causes and risk factors of physical therapists’ burnout.

### Comparison with previous studies

No comparable studies have been conducted on the relationships between burnout and education, mentorship, and self-efficacy in physical therapists in the United States.

### Limitations

Several limitations may have influenced the results of the study. Sampling bias was a possible limitation as respondents who were computer-literate, interested in the topic, or experiencing burnout may have been more likely to have opened and completed the survey. Additionally, a sample of convenience of licensed physical therapists was used, and disproportionately many respondents were employed in Texas (36.05%), which may impact the generalizability of the results.

The onset of COVID-19 is another factor that may have impacted the study results. Changes in the care delivery model, employment status, individual and familial health status, loss of income, and lack of socialization may have contributed to stress, anxiety, and depression and influenced physical therapists’ burnout levels. The burnout rate experienced in our sample, however, was comparable to that experienced by physical therapists prior to the global pandemic [2].

### Generalizability

The results of this study are generalizable as physical therapists from all states were sampled. The respondents were representative in terms of age, race, and sex of the physical therapists currently practicing in the United States [12].

### Conclusion

Burnout appears to be prevalent in the physical therapy profession, as almost half of respondents (49.3%) reported being burned out. This finding is noteworthy as burnout is associated with various negative consequences, including job dissatisfaction, lower levels of empathy, and suboptimal quality of care. Several modifiable factors, including providing or receiving mentorship and greater self-efficacy were associated with lower burnout. Mentorship may contribute to a sense of accomplishment, personal and professional growth. Greater self-efficacy may equip physical therapists with the ability to cope with workplace stress and challenging demands. Organizations should consider measuring burnout with formal instruments or self-reports, as the phenomenon may be pervasive. Investment into mentor and mentee relationships and self-efficacy training may also be rewarding and contribute to lower burnout.

## Figures and Tables

**Figure f2-jeehp-20-27:**
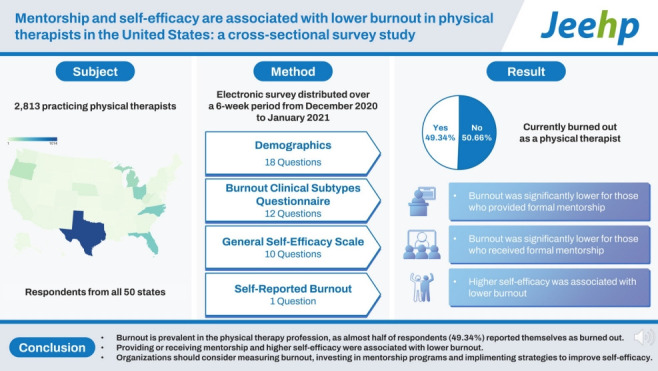


**Table 1.** Demographic data including burnout (BCSQ-12 and self-report) of survey respondents (n=2,813)

Characteristic	Total	Total BCSQ-12		Burned out self-report (%)^a)^
		Median (range)	Mean±SD	
Gender				
Female	1,933 (68.72)	39.00 (12–78)	38.63±11.27	50.34
Male	851 (30.25)	39.00 (12–71)	38.32±12.36	46.30
Other	1 (0.04)			
Prefer not to answer	28 (1.00)	41.50 (18–70)	48.18±12.35	71.43
Age (yr)	44.68±10.55 (24–77)			
Clinical experience (yr)	21.23±20.27 (0–54)			
Race or ethnicity				
American Indian or Alaskan Native	4 (0.14)	42.00 (23–44)	37.75±10.67	50.00
Asian or Pacific Islander	221 (7.86)	43.00 (12–69)	43.38±12.37	55.20
Black or African American	94 (3.34)	38.00 (12–70)	37.98±11.12	51.06
Hispanic or Latino	159 (5.65)	39.00 (15–64)	38.45±11.16	47.80
Multiracial or Biracial	57 (2.03)	42.00 (16–66)	40.33±11.13	54.39
Other/prefer not to answer	24 (0.85)	43.50 (15–84)	44.92±15.56	58.33
White or Caucasian	2,254 (80.13)	39.00 (12–78)	38.16±11.51	48.58
Population of practice area				
Rural (<50,000)	655 (23.28)	39.00 (12–84)	38.71±11.74	45.80
Urban (≥50,000)	2,158 (76.72)	39.00 (12–71)	38.48±11.58	50.42
Employment status				
Full-time	2,170 (77.14)	39.00 (12–84)	38.82±11.65	44.00
Part-time	493 (17.53)	38.00 (12–62)	37.32±11.75	44.62
On-call	150 (5.33)	40.00 (12–61)	38.37±10.57	50.78
Currently burned out as a physical therapist (all respondents)				
No	1,425 (50.66)	33.00 (12–59)	31.74±9.71	
Yes	1,388 (49.34)	45.00 (12–84)	45.52±8.98	

Values are presented as number (%) or mean±SD (range) unless otherwise stated. BCSQ-12, Burnout Clinical Subtypes Questionnaire 12; SD, standard deviation.

a) The burnout percentage was calculated by dividing the number of respondents who reported being burned out by the total number of respondents for each characteristic.

**Table 2.** Practice setting characteristics and burnout (BCSQ-12 and self-report) of survey respondents (n=2,813)

Characteristic	Total	Total BCSQ-12		Burned out self-report (%)^a)^
		Median (range)	Mean±SD	
Practice setting				
Academic	10 (0.36)	33.50 (16–63)	33.90±14.53	20.00
Acute care hospital	404 (14.36)	40.00 (12–71)	38.94±10.82	42.56
Early intervention	16 (0.57)	35.50 (12–52)	34.38±12.42	43.75
Hospital-based outpatient clinic	627 (22.29)	38.00 (12–84)	37.20±11.63	45.45
Home health	430 (15.29)	42.00 (12–71)	41.03±11.68	56.98
Industrial	9 (0.32)	36.00 (14–55)	34.78±14.86	33.33
Military outpatient	18 (0.64)	41.00 (12–69)	40.39±13.71	44.44
Multiple settings or travel	12 (0.43)	42.50 (25–56)	40.67±11.39	58.33
Private outpatient office or group practice	829 (29.47)	39.00 (12–71)	37.16±11.63	48.73
School system	80 (2.84)	35.50 (12–71)	34.75±11.89	27.50
Skilled nursing facility	267 (9.49)	43.00 (16–78)	42.79±10.80	63.30
Sub-acute inpatient rehab hospital	111 (3.95)	40.00 (12–60)	38.50±10.12	54.05
Care delivery model				
In-person patients only	2,301 (81.80)	40.00 (12–78)	38.72±11.61	50.28
Mixed (in-person and remote)	463 (16.46)	39.00 (12–84)	37.86±11.55	45.36
Remote patients only	49 (1.74)	37.00 (12–61)	36.10±12.46	42.86
Average hours of direct patient care per week (hr)	32.94±10.63 (1–60)			

Values are presented as number (%) or mean±SD (range) unless otherwise stated. BCSQ-12, Burnout Clinical Subtypes Questionnaire 12; SD, standard deviation.

a) The burnout percentage was calculated by dividing the number of respondents who reported being burned out by the total number of respondents for each characteristic.

**Table 3.** Professional education, organization memberships, and burnout (BCSQ-12 and self-report) of survey respondents (n=2,813)

Characteristic	Total no. (%)	Total BCSQ-12		Burned out self-report (%)^a)^
		Median (range)	Mean±SD	
Highest PT degree				
Bachelor’s degree (PT)	607 (21.58)	38.00 (12–70)	36.00±11.59	44.15
Master’s degree (MPT)	708 (25.17)	39.00 (12–84)	38.63±11.78	49.01
Doctorate degree (DPT)	1,438 (51.12)	40.00 (12–71)	39.72±11.59	52.71
Terminal degree (PhD, ScD, DSc, EdD or equivalent)	60 (2.13)	33.50 (12–66)	34.53±12.01	25.00
Total hours of continuing education: 3 years (hr)				
0–19	122 (4.34)	41.00 (12–71)	40.45±11.09	50.00
20–39	897 (31.89)	40.00 (12–84)	39.49±11.50	53.51
40–59	859 (30.54)	39.00 (12–78)	38.23±11.46	47.26
60–79	420 (14.93)	39.00 (12–71)	37.75±11.80	48.57
80–99	189 (6.72)	38.00 (12–67)	36.66±11.89	44.44
≥100	326 (11.59)	39.00 (12–69)	38.09±11.92	46.93
Total no. of professional certifications				
0	2,009 (71.42)	39.00 (12–84)	38.67±11.76	49.38
1	643 (22.86)	39.00 (12–69)	38.01±11.27	48.83
2	118 (4.19)	39.00 (14–69)	38.48±11.18	50.00
3	27 (0.96)	42.00 (12–61)	39.37±12.59	51.85
4	8 (0.28)	38.00 (34–54)	42.88±9.09	62.50
≥5	8 (0.28)	44.00 (24–53)	41.88±9.86	50.00
Total no. of professional organization memberships				
0	1,479 (52.58)	40.00 (12–84)	39.00±11.80	52.33
1	818 (29.08)	39.00 (12–69)	38.41±11.04	49.63
2	381 (13.54)	38.00 (12–69)	37.44±11.64	40.94
3	105 (3.73)	38.00 (12–67)	37.44±12.33	37.14
4	17 (0.60)	37.00 (12–59)	35.94±14.57	47.06
≥5	13 (0.46)	38.00 (17–64)	38.46±14.18	38.46

BCSQ-12, Burnout Clinical Subtypes Questionnaire 12; SD, standard deviation; PT, physical therapists.

a) The burnout percentage was calculated by dividing the number of respondents who reported being burned out by the total number of respondents for each characteristic.

**Table 4.** Mentorship experience and burnout (BCSQ-12 and self-report) of survey respondents (n=2,813)

Characteristic	Total no. (%)	Total BCSQ-12		Burned out self-report (%)^a)^
		Median (range)	Mean±SD	
Mentorship received				
None	701 (24.92)	41.00 (12–84)	39.95±12.16	56.49
Informal (training from peers)	1,826 (64.91)	39.00 (12–78)	38.23±11.33	47.26
Formal (residency or fellowship)	286 (10.17)	38.00 (12–66)	37.03±11.78	45.10
Total no. of mentorship experiences received				
0	701 (24.92)	41.00 (12–84)	39.95±12.16	56.49
1	1,973 (70.14)	39.00 (12–78)	38.10±11.42	47.03
2	123 (4.37)	38.00 (12–60)	37.47±11.11	44.72
3	16 (0.57)	40.50 (12–55)	38.38±11.22	56.25
Mentorship provided				
None	596 (21.19)	41.00 (12–84)	39.95±12.16	56.71
Informal (training of peers)	823 (29.26)	40.00 (12–68)	38.27±11.34	47.87
Formal (student, residency, or fellowship)	1,394 (49.56)	39.00 (12–78)	37.03±11.78	47.06
Total no. of mentorship experiences provided				
0	596 (21.19)	41.00 (12–84)	40.27±11.88	56.71
1	1,109 (39.42)	39.00 (12–71)	38.40±11.86	47.16
2	872 (31.00)	39.00 (12–78)	38.07±11.09	47.71
3	189 (6.72)	36.00 (12–61)	35.62±11.44	43.92
4	39 (1.39)	41.00 (18–61)	40.13±9.95	64.10
5	8 (0.28)	46.00 (20–48)	40.00±10.70	37.50

BCSQ-12, Burnout Clinical Subtypes Questionnaire 12; SD, standard deviation.

a) The burnout percentage was calculated by dividing the number of respondents who reported being burned out by the total number of respondents for each characteristic.

## Footnotes

**Data availability**
Data files are available from Harvard Dataverse: https://doi.org/10.7910/DVN/XAPD4U Dataset 1. Raw response data from 2,813 physical therapists from the United States.

Dataset 2. Coded response data.

## Supplementary materials

Supplementary files are available from Harvard Dataverse: https://doi.org/10.7910/DVN/XAPD4U

Supplement 1. Survey questionnaire on the prevalence of burnout in physical therapists in the United States and relationships between burnout and education, mentorship, and self-efficacy.

Supplement 2. Audio recording of the abstract.
