# Supplementary material for: Mentorship and self-efficacy are associated with lower burnout in physical therapists in the United States: a cross-sectional survey study
Source: J Educ Eval Health Prof. 2023 Sep 27;20:27. doi: 10.3352/jeehp.2023.20.27 (PMC10632729; doi:10.3352/jeehp.2023.20.27)
Supplement: Supplementary file 3 — Supplement 1. Survey questionnaire on the prevalence of burnout in physical therapists in the United States and relationships between burnout and education, mentorship, and self-efficacy. [file jeehp-20-27-suppl.docx]

**Supplement 1.** Survey questionnaire on the prevalence of burnout in physical therapists in the United States and relationships between burnout and education, mentorship, and self-efficacy

**Survey instrument**

**1. Are you currently evaluating and treating patients as a licensed physical therapist?**

○ Yes

○ No

**2. What is your age?**

○ 18–80 years (drop down menu)

**3. What is your gender?**

○ Female

○ Male

○ Prefer not to answer

○ Other (please specify–write in box)

**4. Which race or ethnicity best describes you?** (Please choose only one).

○ American Indian or Alaskan Native

○ Asian or Pacific Islander

○ Black or African American

○ Hispanic or Latino

○ White or Caucasian

○ Multiracial or Biracial

○ Other (please specify–write in box)

**5. What state do you practice in?**

○ List of States (drop down menu)

**6. Which of the following best describes the area you practice in?**

○ Population <50,000 (rural)

○ Population >50,000 (urban)

**7. How many years of clinical experience do you have working as a physical therapist?**

○ 0–60 years (drop down menu)

**8. Which best describes your current employment status as a licensed physical therapist?**

○ Full-time

○ Part-time

○ On-call

○ Other (please specify–write in box)

**9. On average, how many hours per week did you provide direct patient care as a physical therapist over the last 3 years?**

○ 0–60 hours (drop down menu). An answer of “0 hours” disqualifies respondent.

**10. Which best describes your current physical therapy practice setting where you most frequently practiced in the last 3 years?**

○ Acute care hospital

○ Sub-acute inpatient rehab hospital

○ Health system or hospital-based outpatient clinic

○ Private outpatient office or group practice

○ Skilled nursing facility/extended care facility

○ Home health

○ School system

○ Other (please specify–write in box)

**11. Which best describes your current physical therapy care delivery model?**

○ In-person patients only (clinical setting)

○ Remote patients only (telerehabilitation)

○ Mixed (in-person and telerehabilitation)

○ Other (please specify–write in box)

**12. What is the highest physical therapy related degree you have completed?**

○ Bachelor (PT)

○ Master (MPT)

○ Doctorate Degree (DPT)

○ Terminal Degree (PhD, ScD, DSc, EdD or equivalent)

**13. Select all the job roles you have held in the last 3 years?** (select all that apply–checkbox)

○ Clinician

○ Clinical instructor (students)

○ Supervisor/director/administrative

○ Practice owner

○ Clinical educator (residency, fellowship)

○ Faculty (adjunct faculty, full-time faculty)

○ Researcher

○ Other (please specify–write in box)

**14. What are the total number of hours of continuing education you have completed in the past 3 years (2018–2020)?**

○ 0–19 hours

○ 20–39 hours

○ 40–69 hours

○ 60–79 hours

○ 80–99 hours

○ 100 hours or greater

**15.** **How many professional certifications recognized by the American Physical Therapy Association (APTA) have you completed?** (cardiovascular & pulmonary, clinical electrophysiology, geriatrics, neurology, oncology, orthopedics, pediatrics, sports, women’s health, wound management, residency training, fellowship training)

○ 0

○ 1

○ 2

○ 3

○ 4

○ 5 or greater

**16. How many professional organizations related to physical therapy are you a member of?** (American Physical Therapy Association, American Academy of Orthopedic Manual Physical Therapists, American Society of Hand Therapists, National Strength and Conditioning Association, etc.)

○ 0

○ 1

○ 2

○ 3

○ 4

○ 5 or greater

**17. Select the type(s) of post-professional clinical mentorship you have received throughout your career?** (select all that apply–checkbox)

○ None

○ Informal mentorship (training from peers)

○ Formal residency program

○ Formal fellowship program

○ Other (please specify–write in box)

**18. Select the type(s) of post-professional clinical mentorship you have provided in the last 3 years?** (select all that apply–checkbox)

○ None

○ Informal mentorship (training of peers)

○ Instructor of students in a clinical setting

○ Instructor of students in an academic setting

○ Formal residency program mentor

○ Formal fellowship program mentor

○ Other (please specify – write in box)

**BCSQ-12 (Burnout Clinical Subtypes Questionnaire 12; burnout)**

**19. I think the dedication I invest in my work is more than what I should for my health.**

○ Totally disagree

○ Strongly disagree

○ Disagree

○ Unsure

○ Agree

○ Strongly agree

○ Totally agree

**20. I would like to be doing another job that is more challenging for my abilities.**

○ Totally disagree

○ Strongly disagree

○ Disagree

○ Unsure

○ Agree

○ Strongly agree

○ Totally agree

**21. When things at work don’t turn out as well as they should, I stop trying.**

○ Totally disagree

○ Strongly disagree

○ Disagree

○ Unsure

○ Agree

○ Strongly agree

○ Totally agree

**22. I neglect my personal life when I pursue important achievements in my work.**

○ Totally disagree

○ Strongly disagree

○ Disagree

○ Unsure

○ Agree

○ Strongly agree

○ Totally agree

**23. I feel that my work is an obstacle to the development of my abilities.**

○ Totally disagree

○ Strongly disagree

○ Disagree

○ Unsure

○ Agree

○ Strongly agree

○ Totally agree

**24. I give up in response to difficulties in my work.**

○ Totally disagree

○ Strongly disagree

○ Disagree

○ Unsure

○ Agree

○ Strongly agree

○ Totally agree

**25. I risk my health when I pursue good results in my work.**

○ Totally disagree

○ Strongly disagree

○ Disagree

○ Unsure

○ Agree

○ Strongly agree

○ Totally agree

**26. I would like to be doing another job where I can better develop my talents.**

○ Totally disagree

○ Strongly disagree

○ Disagree

○ Unsure

○ Agree

○ Strongly agree

○ Totally agree

**27. I give up in the face of any difficulties in my work tasks.**

○ Totally disagree

○ Strongly disagree

○ Disagree

○ Unsure

○ Agree

○ Strongly agree

○ Totally agree

**28. I overlook my own needs to fulfill work demands.**

○ Totally disagree

○ Strongly disagree

○ Disagree

○ Unsure

○ Agree

○ Strongly agree

○ Totally agree

**29. My work doesn’t offer me opportunities to develop my abilities.**

○ Totally disagree

○ Strongly disagree

○ Disagree

○ Unsure

○ Agree

○ Strongly agree

○ Totally agree

**30. When the effort I invest in work is not enough, I give in.**

○ Totally disagree

○ Strongly disagree

○ Disagree

○ Unsure

○ Agree

○ Strongly agree

○ Totally agree

**GSES (General Self-Efficacy Scale; self-efficacy)**

**31. I can always manage to solve difficult problems if I try hard enough.**

○ Not true at all

○ Barely true

○ Moderately true

○ Exactly true

**32. If someone opposes me, I can find the means and ways to get what I want.**

○ Not true at all

○ Barely true

○ Moderately true

○ Exactly true

**33. It is easy for me to stick to my aims and accomplish my goals.**

○ Not true at all

○ Barely true

○ Moderately true

○ Exactly true

**34. I am confident that I could deal efficiently with unexpected events.**

○ Not true at all

○ Barely true

○ Moderately true

○ Exactly true

**35. Thanks to my resourcefulness, I know how to handle unforeseen situations.**

○ Not true at all

○ Barely true

○ Moderately true

○ Exactly true

**36. I can solve most problems if I invest the necessary effort.**

○ Not true at all

○ Barely true

○ Moderately true

○ Exactly true

**37. I can remain calm when facing difficulties because I can rely on my coping abilities.**

○ Not true at all

○ Barely true

○ Moderately true

○ Exactly true

**38. When I am confronted with a problem, I can usually find several solutions.**

○ Not true at all

○ Barely true

○ Moderately true

○ Exactly true

**39. If I am in trouble, I can usually think of a solution.**

○ Not true at all

○ Barely true

○ Moderately true

○ Exactly true

**40. I can usually handle whatever comes my way.**

○ Not true at all

○ Barely true

○ Moderately true

○ Exactly true

**41. Do you currently consider yourself to be burned out as a physical therapist?**

○ Yes

○ No

Thank you for completing the survey.
